# Supplementary material for: Replication fork slowing and stalling are distinct, checkpoint-independent consequences of replicating damaged DNA
Source: PLoS Genet. 2017 Aug 14;13(8):e1006958. doi: 10.1371/journal.pgen.1006958 (PMC5570505; doi:10.1371/journal.pgen.1006958)
Supplement: S1 Fig — yFS105 asynchronous, log-phase cultures were untreated or treated with 3.5mM MMS or 16.5μM bleomycin for 1 hour. (A) Estimation of lesion density caused by MMS. Genomic DNA was isolated from untreated and MMS treated samples and digested with EcoRV and ScaI and heated at 65°C for 2 hours. The resulting satellite bands were analyzed by alkaline agarose gel electrophoresis. (B) Estimation of lesion density caused by bleomycin. Agarose plugs were prepared from untreated and bleomycin treated samples. The plugs were digested with EcoRV and ScaI and the resulting satellite bands were analyzed by neutral agarose gel electrophoresis. The DNA in Peak1, which represents the exclusion volume of the gel, is assumed to average ~20kb. The lanes were quantified using ImageJ software. Lesion density was inferred by assuming a Poisson distribution of lesions and calculating the frequency of lesions predicted to result in the observed fraction of molecules with no breaks. (PDF) [file pgen.1006958.s001.pdf]

Figure S1

A Estimation of lesion density caused by MMS

Alkaline agarose gel electrophoresis

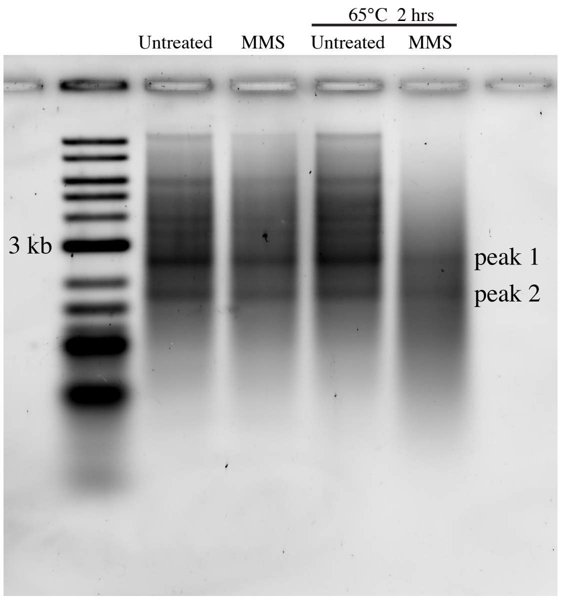

ImageJ quantification of untreated and MMS-treated samples heated at 65°C

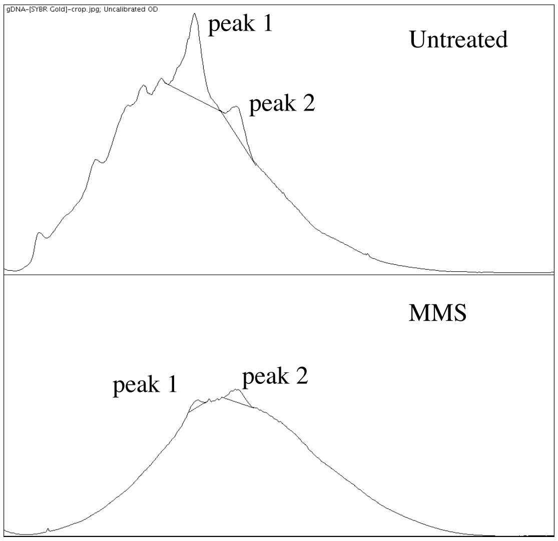

	fraction intensity over total intensity of the lane Untreated	MMS	fraction of peaks remaining	DNA size in kb	breaks per kb
Peak 1	0.042	0.0027	0.64	2.6	1.1
Peak 2	0.012	0.0077	0.63	1.8	0.3

B Estimation of lesion density caused by bleomycin

Neutral agarose gel electrophoresis

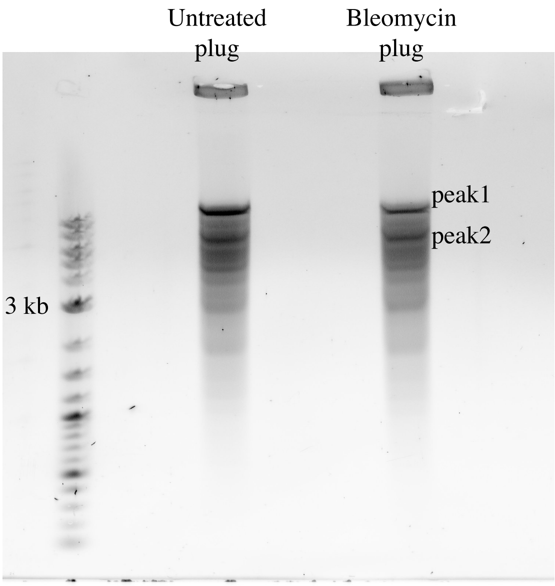

ImageJ quantification of untreated and bleomycin-treated samples

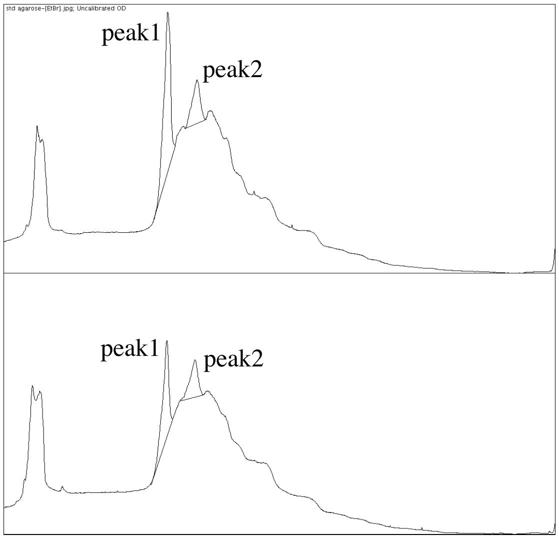

	fraction intensity over total intensity of the lane Untreated	Bleomycin	fraction of peaks remaining	DNA size in kb	breaks per Mb
Peak 1	0.046	0.028	0.61	~20	25
Peak 2	0.015	0.014	0.93	7.0	14

Figure S1: Estimation of lesion density from MMS- and bleomycin-treated samples. yFS105 asynchronous, log-phase cultures were untreated or treated with 3.5mM MMS or 16.5μM bleomycin for 1 hour. (A) Estimation of lesion density caused by MMS. Genomic DNA was isolated from untreated and MMS treated samples and digested with EcoRV and ScaI and heated at 65°C for 2 hours. The restriction fragments were analyzed by alkaline agarose gel electrophoresis. (B) Estimation of lesion density caused by bleomycin. Agarose plugs were prepared from untreated and bleomycin treated samples. The plugs were digested with EcoRV and ScaI and the restriction fragments were analyzed by neutral agarose gel electrophoresis. The DNA in Peak1, which represents the exclusion volume of the gel, is assumed to average ~20 kb. The lanes were quantified using ImageJ software.
