# Supplementary material for: Replication fork slowing and stalling are distinct, checkpoint-independent consequences of replicating damaged DNA
Source: PLoS Genet. 2017 Aug 14;13(8):e1006958. doi: 10.1371/journal.pgen.1006958 (PMC5570505; doi:10.1371/journal.pgen.1006958)
Supplement: S2 Fig — (A) Estimation of apparent stall rate from unambiguous events. (B) Estimation of stall rate from all events–ambiguous as well as unambiguous events. (PDF) [file pgen.1006958.s002.pdf]

Figure S2: Schematic explanation of stall rate estimation

A

Estimation of apparent stall rate

Apparent stall rate was calculated from the unambiguous stall events (RUG and GUR) as explained in the text.

Signature for stalled forks

Signature for unstalled forks

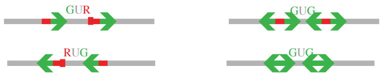

R - red
G - green
U - unlabeled
st - apparent stall rate
n - number of events in the entire dataset

$$st = \frac{n(RUG) + n(GUR)}{n(GUG) + n(RUG) + n(GUR)}$$

The stalled forks are included in the denominator beacuse every stall leads to loss of one fork.
For example, consider an origin (GRG) on a fiber. If one of its fork is stalled then it will appear as GR or RG and
we would calculate the stall rate as 50% since one fork is stalled however ideally there should be two (one fork which we can visualize and one which is stalled).

B

Estimation of stall rate from all events - ambiguous as well as unambiguous events

? - ambiguous stall events

	Description for the bold-italicized track	Probability of the bold-italicized red track having a stalled fork	Probability of the bold-italicized red track being an origin with two stalled forks	Total number of stalled forks	Total number of unstalled forks	Total number of ongoing forks
GU***R***G	red track is an origin with a stalled leftward fork	1	-	n(GURG)	n(GURG)	2*n(GURG)
U***R***G ?	red track could be an origin with a stalled leftward fork or just an elongating rightward fork	st	-	n(URG)*st	n(URG)*(1-st)	n(URG)*(1-st) + 2*st*n(URG)
RU***R***G ? ?	elongating forks from an origin in the center or two origins whose inner forks have stalled	st^2	-	n(RURG)*st^2	n(RURG)*(1-st^2)	n(RURG)*(1-st^2) + 2*st^2*n(RURG)
G***R***UG	red track is an origin with a stalled rightward fork	1	-	n(GRUG)	n(GRUG)	2*n(GRUG)
? G***R***U	red track could be an origin with a stalled rightward fork or just an elongating leftward fork	st	-	n(GRU)*st	n(GRU)*(1-st)	n(GRU)*(1-st) + 2*st*n(GRU)
G***R***UR ? ?	elongating forks from an origin in the center or two origins whose inner forks have stalled	st^2	-	n(GRUR)*st^2	n(GRUR)*(1-st^2)	n(GRUR)*(1-st^2) + 2*st^2*n(GRUR)
GU***R***U ?	red track could be an elongating fork with a stalled leftward fork or an origin with two stalls	1-st	st	n(GURU)*(1-st) + 2*n(GURU)*st	-	n(GURU)*(1-st) + 2*n(GURU)*st
? U***R***UG	red track could be an elongating fork with a stalled rightward fork or an origin with two stalls	1-st	st	n(URUG)*(1-st) + 2*n(URUG)*st	-	n(URUG)*(1-st) + 2*n(URUG)*st
RU***R***U ? ?	red track could be a termination event or an origin with two stalls or an elongating fork with stall on either side	st + st^2 - 2*st^3	st^3	n(RURU)*(st + st^2 - 2*st^3) + 2*n(RURU)*st^3	2*n(RURU)*(1 - st - st^2 + st^3)	n(RURU)*(st + st^2 - 2*st^3) + 2*n(RURU)*st^3 + 2*n(RURU)*(1 - st - st^2 + st^3)
? ? U***R***UR	red track could be a termination event or an origin with two stalls or an elongating fork with stall on either side	st + st^2 - 2*st^3	st^3	n(URUR)*(st + st^2 - 2*st^3) + 2*n(URUR)*st^3	2*n(URUR)*(1 - st - st^2 + st^3)	n(URUR)*(st + st^2 - 2*st^3) + 2*n(URUR)*st^3 + 2*n(URUR)*(1 - st - st^2 + st^3)
RU***R***UR ? ?	red track could be a termination event or an origin with two stalls or an elongating fork with stall on either side	2*(st^2 - st^4)	st^4	2*n(RURUR)*(st^2 - st^4) + 2*n(RURUR)*st^4	2*n(RURUR)*(1 - 2*st^2 + st^4)	2*n(RURUR)*(st^2 - st^4) + 2*n(RURUR)*st^4 + 2*n(RURUR)*(1 - 2*st^2 + st^4)
? RU***R***UG	red track could be an elongating fork with a stalled rightward fork or could be an origin with two stalls	1-st^2	st^2	n(RURUG)*(1-st^2) + 2*n(RURUG)*st^2	-	n(RURUG)*(1-st^2) +2*n(RURUG)*st^2
GU***R***UR ?	red track could be an elongating fork with a stalled leftward fork or could be an origin with two stalls	1-st^2	st^2	n(GURUR)*(1-st^2) + 2*n(GURUR)*st^2	-	n(GURUR)*(1-st^2) + 2*n(GURUR)*st^2
GU***R***UG	red track is an origin with both its forks stalled	-	1	2*n(GURUG)	-	2*n(GURUG)
U***R***U ? ?	red track could be a termination event or an origin with two stalls or an elongating fork with stall on either side	2*(st - st^2)	st^2	2*n(URU)*(st - st^2) + 2*n(URU)*st^2	2*n(URU)*(1 - 2*st + st^2)	2*n(URU)*(st - st^2) + 2*n(URU)*st^2 + 2*n(URU)*(1 - 2*st + st^2)
G***R***G	red track is an origin with two forks	-	-	-	2*n(GRG)	2*n(GRG)

$$\text{stall rate} = \frac{\text{total number of stalled forks}}{\text{total number of ongoing forks}}$$

Figure S2: **Schematic explanation of stall rate estimation**. (A) Estimation of apparent stall rate from unambiguous events. (B) Estimation of stall rate from all events – ambiguous as well as unambiguous events.
