# Supplementary material for: Replication fork slowing and stalling are distinct, checkpoint-independent consequences of replicating damaged DNA
Source: PLoS Genet. 2017 Aug 14;13(8):e1006958. doi: 10.1371/journal.pgen.1006958 (PMC5570505; doi:10.1371/journal.pgen.1006958)
Supplement: S3 Fig — Wild-type (yFS940) and cds1Δ (yFS941) cells were synchronized and released into S phase with 3.5 mM MMS, 0.5 μM, 1 μM, 2 μM 4NQO or left untreated. S-phase progression was monitored by taking samples every 20 minutes for flow cytometry. (PDF) [file pgen.1006958.s003.pdf]

Figure S3

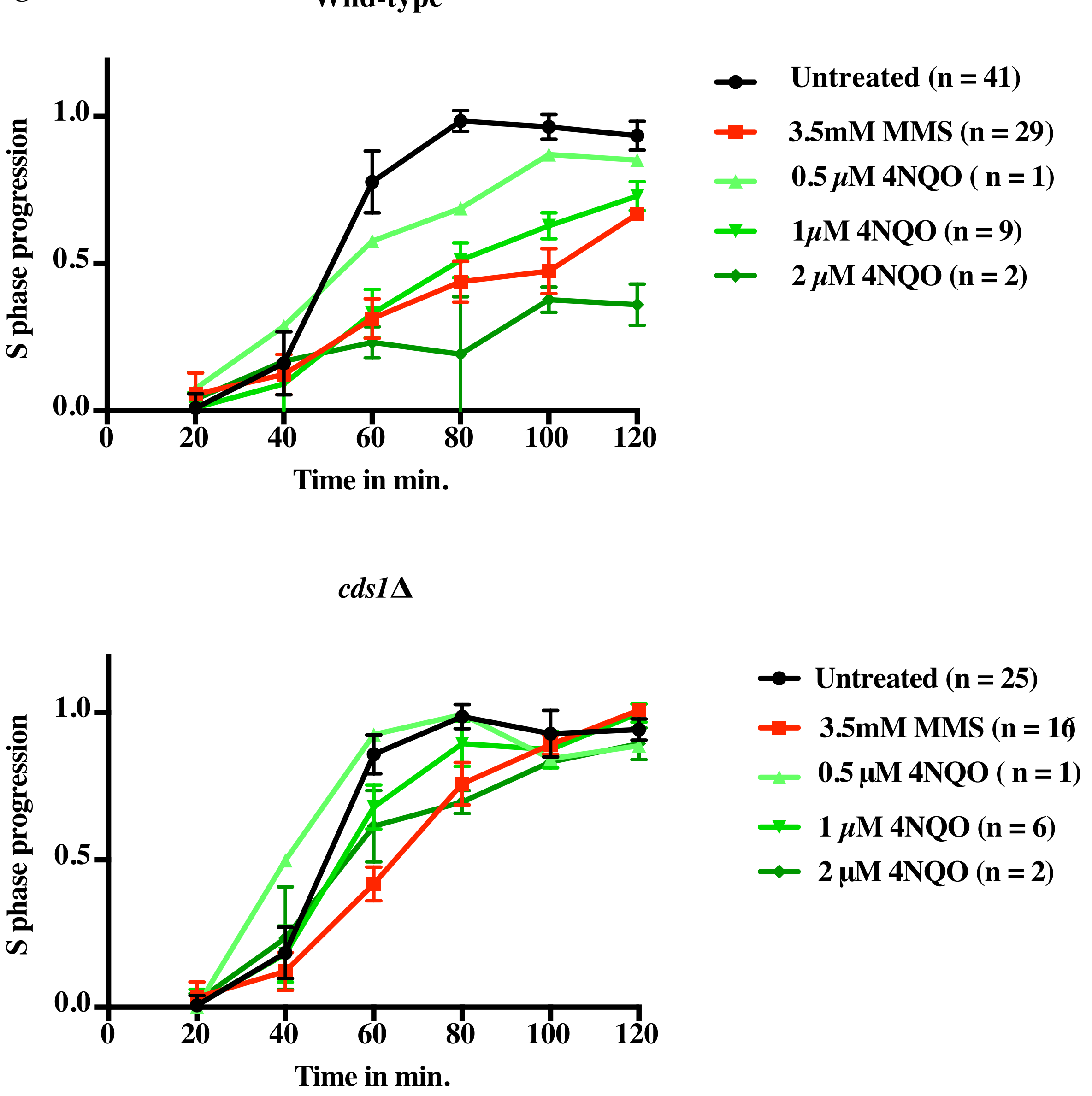

Figure S3:Titration of 4NQO. Wild-type (ySF940) and *cds1*Δ (yFS941) cells were synchronized and released into S phase with 3.5 mM MMS, 0.5 μM, 1 μM, 2μM 4NQO or left untreated. S-phase progression was monitored by taking samples every 20 minutes for flow cytometry.
