# Supplementary material for: Replication fork slowing and stalling are distinct, checkpoint-independent consequences of replicating damaged DNA
Source: PLoS Genet. 2017 Aug 14;13(8):e1006958. doi: 10.1371/journal.pgen.1006958 (PMC5570505; doi:10.1371/journal.pgen.1006958)
Supplement: S4 Fig — Wild-type (yFS940) and cds1Δ (yFS941) cells were synchronized and released into S phase with different concentrations of bleomycin 16.5 μM, 23.79 μM, 47.9 μM or left untreated. S phase progression was monitored by taking samples every 20 minutes for flow cytometry. (PDF) [file pgen.1006958.s004.pdf]

## Figure S4

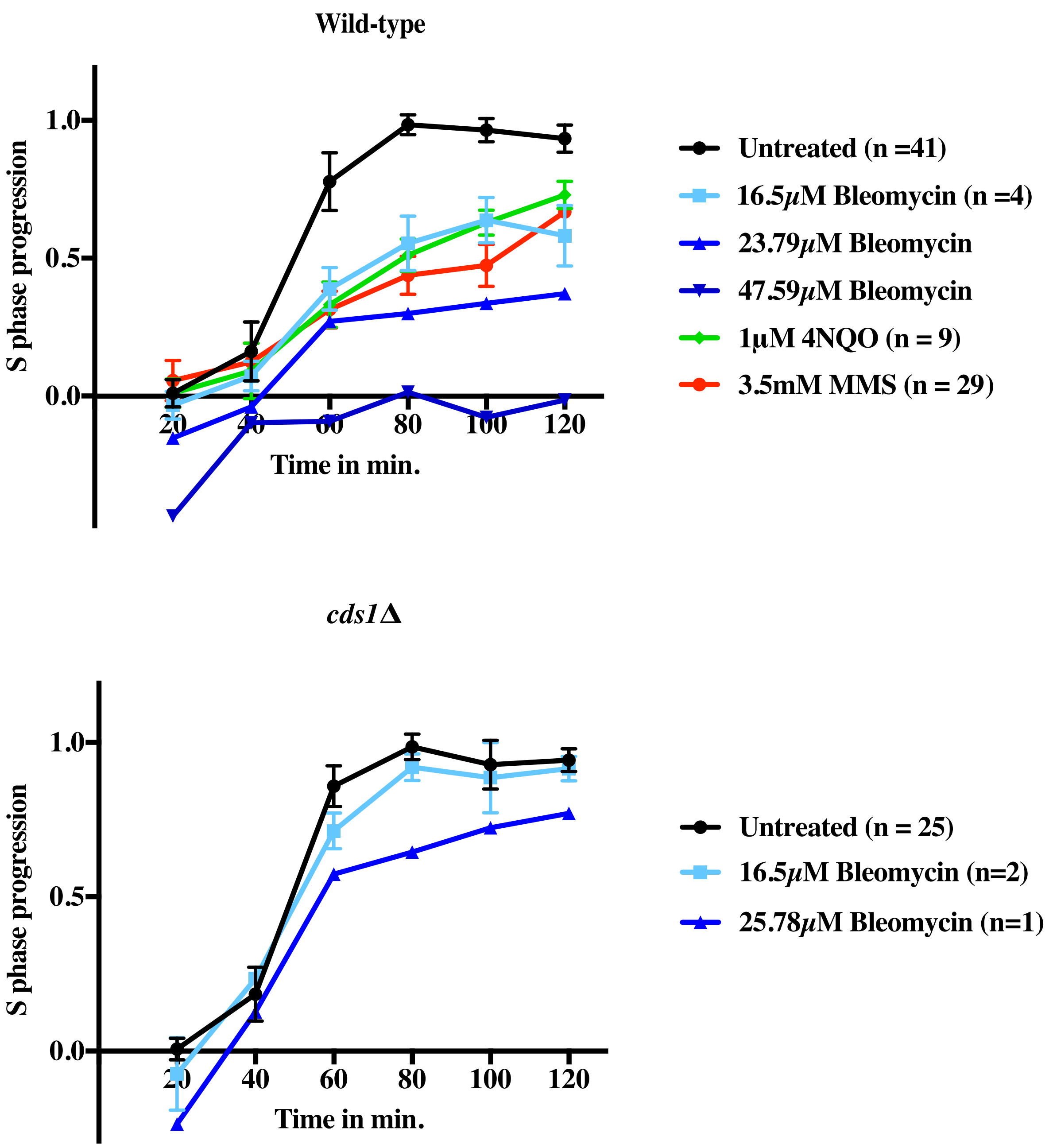


**Figure S4: Titration of Bleomycin.** Wild-type (yFS940) and *cds1*Δ (yFS941) cells were synchronized and released into S phase with different concentrations of bleomycin 16.5μM, 23.79μM, 47.9 μM or left untreated. S phase progression was monitored by taking samples every 20 minutes for flow cytometry.
