# Supplementary material for: Replication fork slowing and stalling are distinct, checkpoint-independent consequences of replicating damaged DNA
Source: PLoS Genet. 2017 Aug 14;13(8):e1006958. doi: 10.1371/journal.pgen.1006958 (PMC5570505; doi:10.1371/journal.pgen.1006958)
Supplement: S5 Fig — WT (yFS940) (A) and cds1Δ (yFS941) (B) cells were synchronized in G1 phase using cdc10-M17 temperature sensitive allele followed by elutriation. Elutriated G1 cells were released into permissive temperature untreated or treated with 3.5 mM MMS or 1 μM 4NQO or 16.5 μM Bleomycin. (PDF) [file pgen.1006958.s005.pdf]

Figure S5

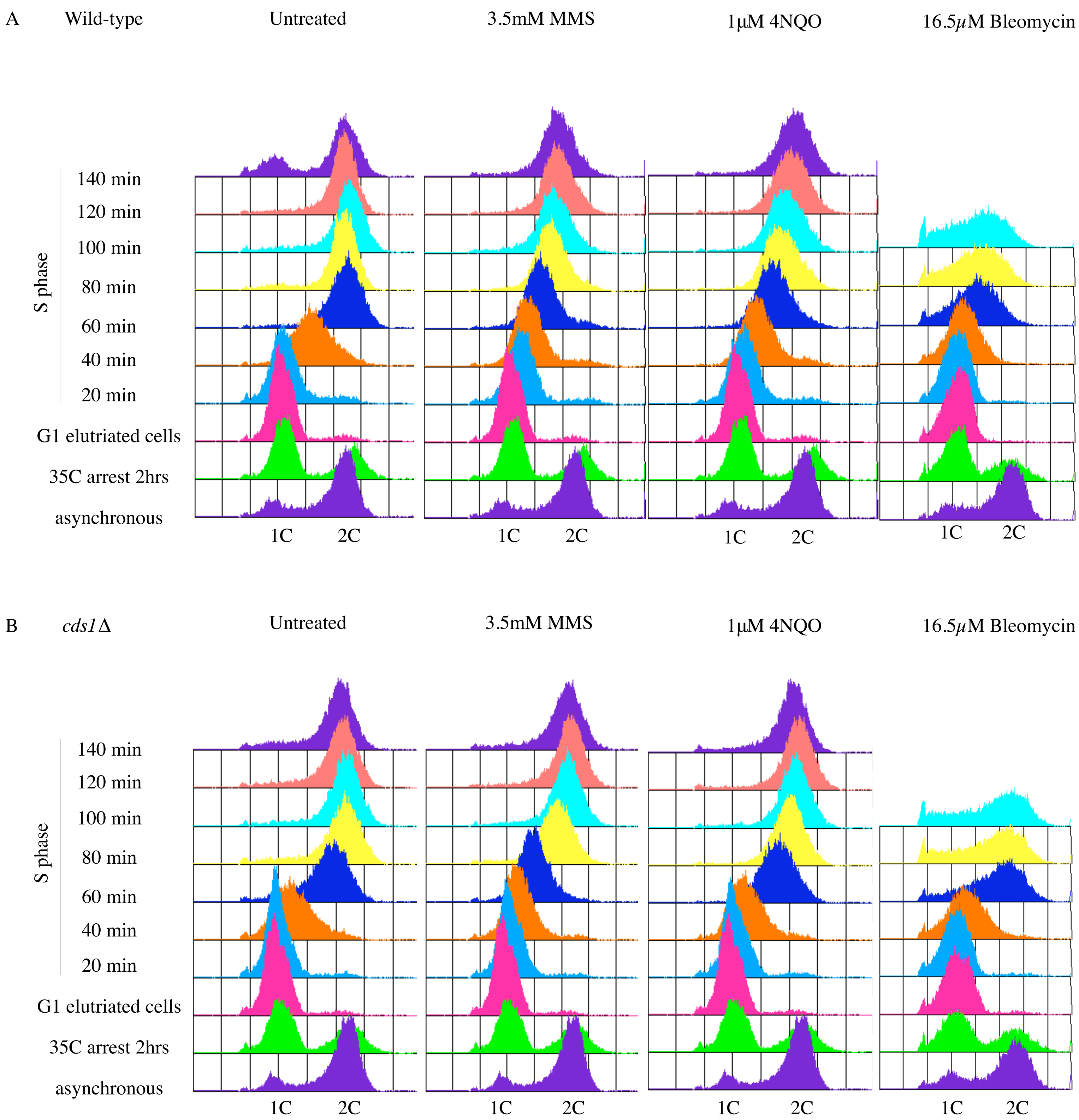

Figure S5: S phase progression by FACS. WT (yFS940) (A) and *cds1*Δ (yFS941) (B) cells were synchronized in G1 phase using cdc10-M17 temperature sensitive allele followed by elutriation. Elutriated G1 cells were released into permissive temperature untreated or treated with 3.5mM MMS or 1μM 4NQO or 16.5μM Bleomycin.
