# Supplementary figures and images for: Replication fork slowing and stalling are distinct, checkpoint-independent consequences of replicating damaged DNA
Source: PLoS Genet. 2017 Aug 14;13(8):e1006958. doi: 10.1371/journal.pgen.1006958 (PMC5570505; doi:10.1371/journal.pgen.1006958)

Figure S6

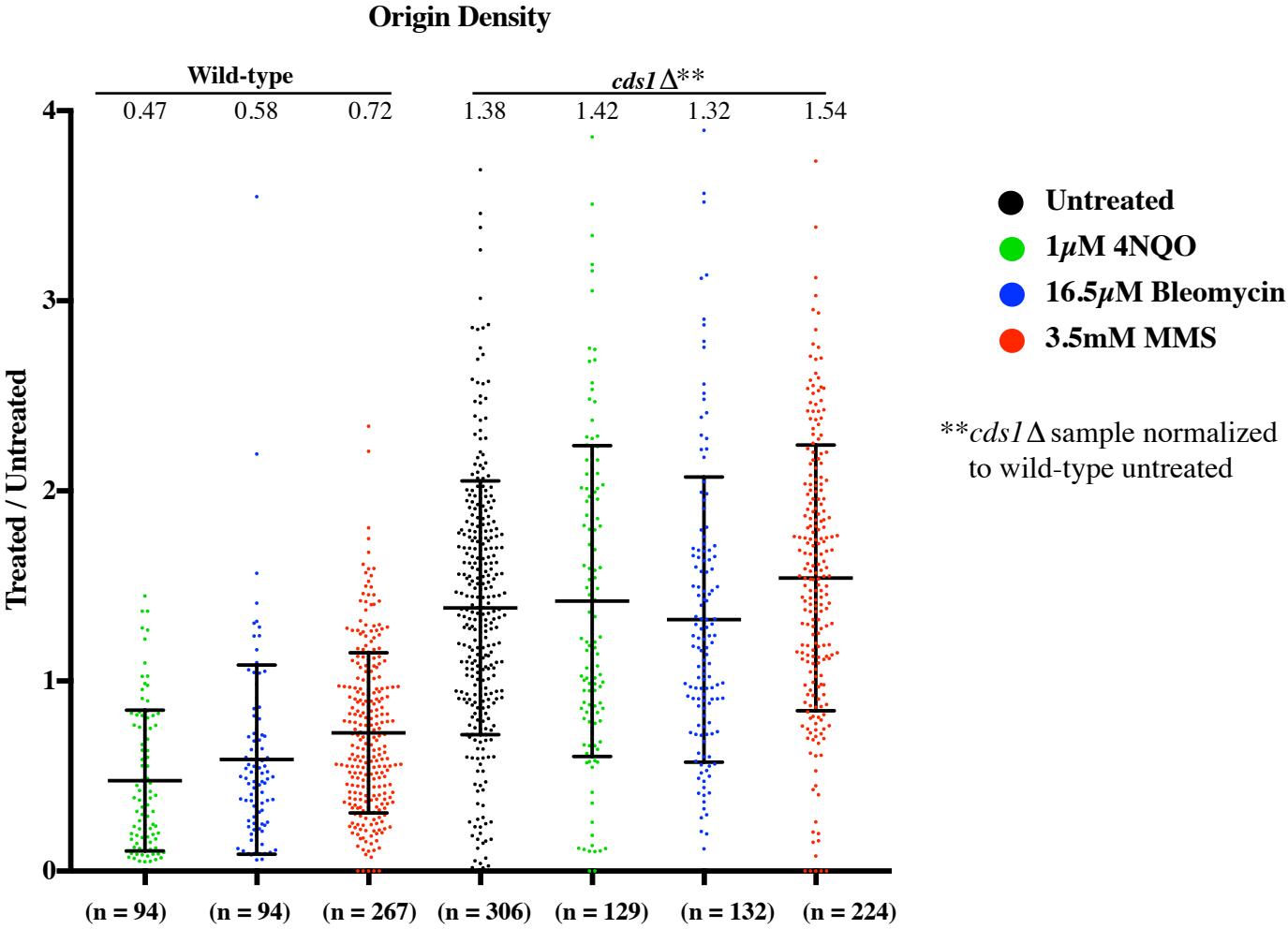

Figure S6: Origin density from *cds1*Δ sample normalized to wild-type untreated sample.

Supplement: S6 Fig — (PDF) [file pgen.1006958.s006.pdf]
