# Supplementary material for: Replication fork slowing and stalling are distinct, checkpoint-independent consequences of replicating damaged DNA
Source: PLoS Genet. 2017 Aug 14;13(8):e1006958. doi: 10.1371/journal.pgen.1006958 (PMC5570505; doi:10.1371/journal.pgen.1006958)
Supplement: S7 Fig — (PDF) [file pgen.1006958.s007.pdf]

Figure S7

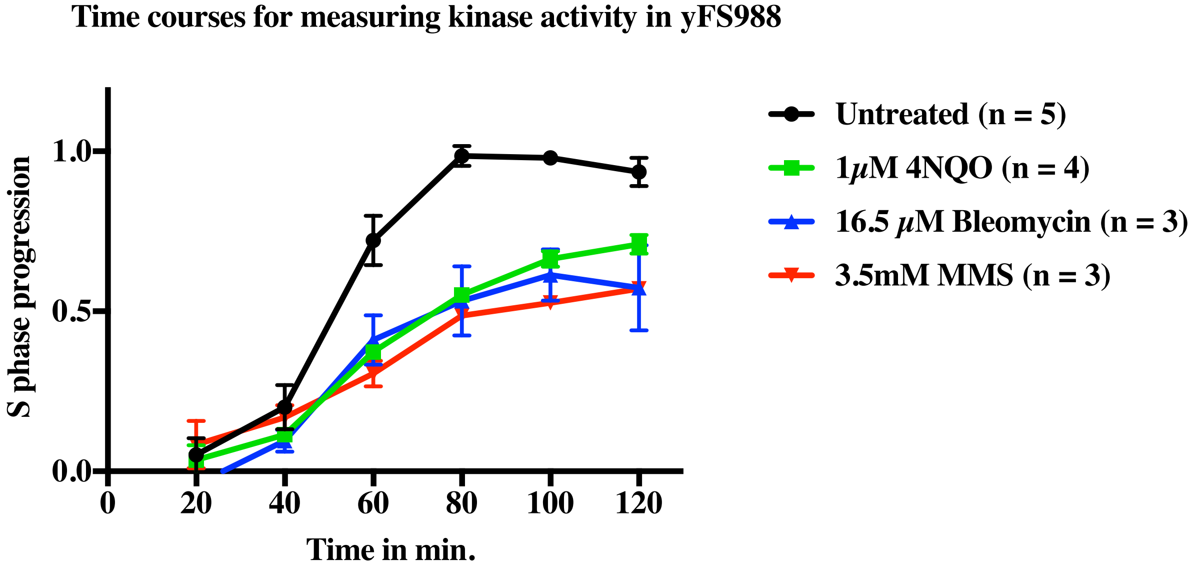

Figure S7: S phase progression by FACS of time courses used for kinase activity measurement
