# Supplementary material for: Replication fork slowing and stalling are distinct, checkpoint-independent consequences of replicating damaged DNA
Source: PLoS Genet. 2017 Aug 14;13(8):e1006958. doi: 10.1371/journal.pgen.1006958 (PMC5570505; doi:10.1371/journal.pgen.1006958)
Supplement: S8 Fig — For each time course, Cds1 western was done to estimate the amount of Cds1 pulled down in each condition. Kinase assay reactions were run normalized to the amount of Cds1 pull down. Signal from each lane of the kinase assay gel was normalized to the asynchronous MMS sample to get the normalized kinase activity. (PDF) [file pgen.1006958.s008.pdf]

Figure S8

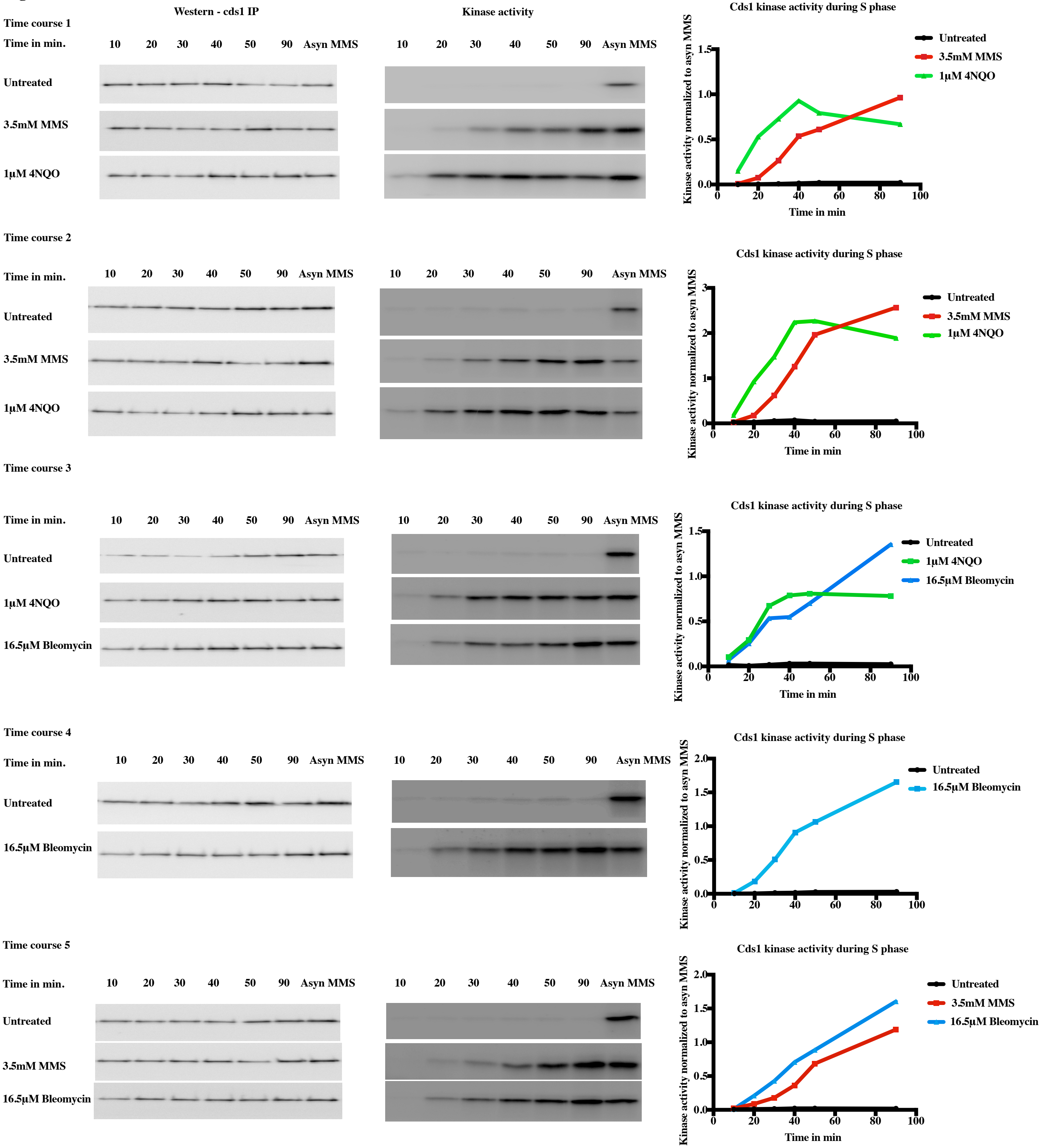

Figure S8: Kinase assay to measure Cds1 activation in 5 time courses. For each time course, Cds1 western was done to estimate the amount of Cds1 pulled down in each condition. Kinase assay reactions were run normalized to the amount of Cds1 pull down. Signal from each lane of the kinase assay gel was normalized to the asynchronous MMS sample to get the normalized kinase activity
