# Supplementary material for: Replication fork slowing and stalling are distinct, checkpoint-independent consequences of replicating damaged DNA
Source: PLoS Genet. 2017 Aug 14;13(8):e1006958. doi: 10.1371/journal.pgen.1006958 (PMC5570505; doi:10.1371/journal.pgen.1006958)
Supplement: S9 Fig — Fork rate distribution in wild-type and cds1Δ cells untreated or treated with MMS from a single experiment at time points 1 and 3, respectively (A and C) and from two experiments at time points 2 and 4, respectively (B and D). For the exact timings refer to S1 Table. Each distribution of fork rate was fit to a Gaussian curve. Mean fork rate was obtained from the fit. (PDF) [file pgen.1006958.s009.pdf]

Figure S9

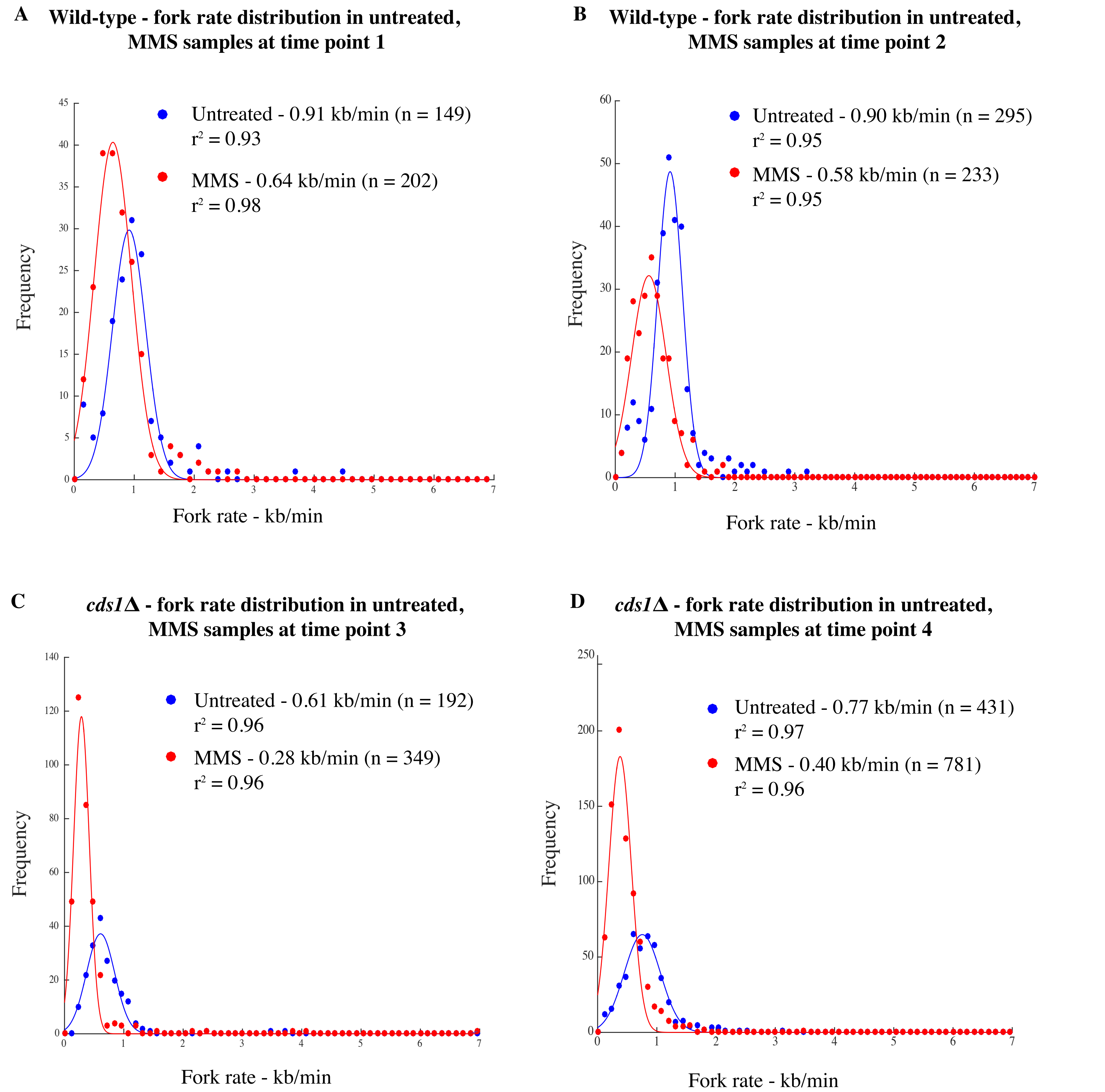

Figure S9: Fork rate distribution. Fork rate distribution in wild-type and *cds1*Δ cells untreated or treated with MMS from a single experiment at time points 1 and 3, respectively (A and C) and from two experiments at time points 2 and 4, respectively (B and D). For the exact timings refer to Table S1. Each distribution of fork rate was fit to a Gaussian curve. Mean fork rate was obtained from the fit.
