# Supplementary material for: Replication fork slowing and stalling are distinct, checkpoint-independent consequences of replicating damaged DNA
Source: PLoS Genet. 2017 Aug 14;13(8):e1006958. doi: 10.1371/journal.pgen.1006958 (PMC5570505; doi:10.1371/journal.pgen.1006958)
Supplement: S10 Fig — (PDF) [file pgen.1006958.s010.pdf]

Figure S10

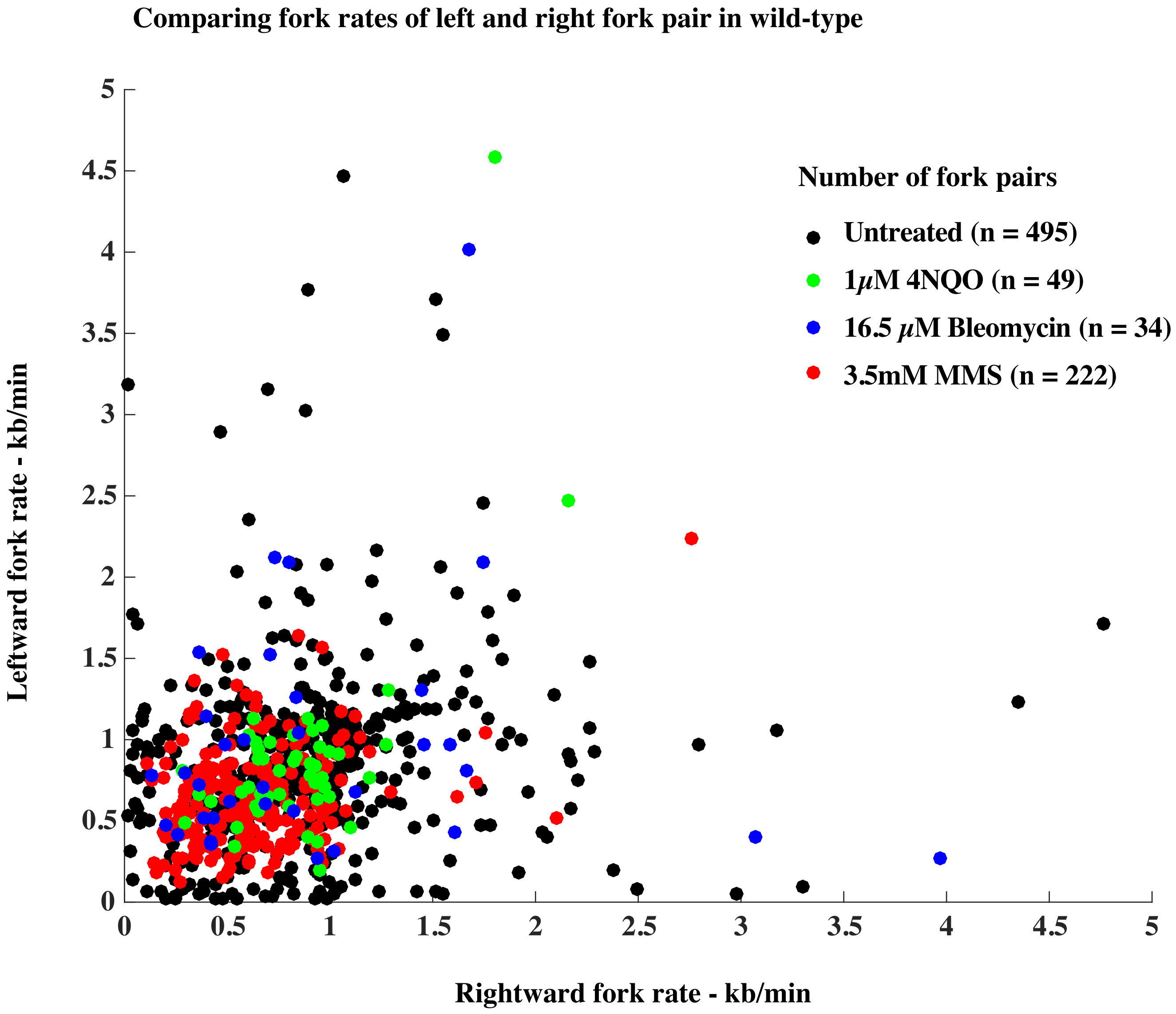

Figure S10: Comparing fork rates of left and right fork pairs in wild-type (yFS940) does not show increased asymmetry in treated samples as compared to untreated sample.
