# Supplementary material for: Replication fork slowing and stalling are distinct, checkpoint-independent consequences of replicating damaged DNA
Source: PLoS Genet. 2017 Aug 14;13(8):e1006958. doi: 10.1371/journal.pgen.1006958 (PMC5570505; doi:10.1371/journal.pgen.1006958)
Supplement: S11 Fig — Length of fibers from wild-type 4NQO (A) and MMS (B) samples from a single experiment. Approximately 25 Mb of total DNA was collected for each sample (S1 Table). (PDF) [file pgen.1006958.s011.pdf]

Figure S11

A **Wild-type - fiber length distribution in untreated and 4NQO sample**

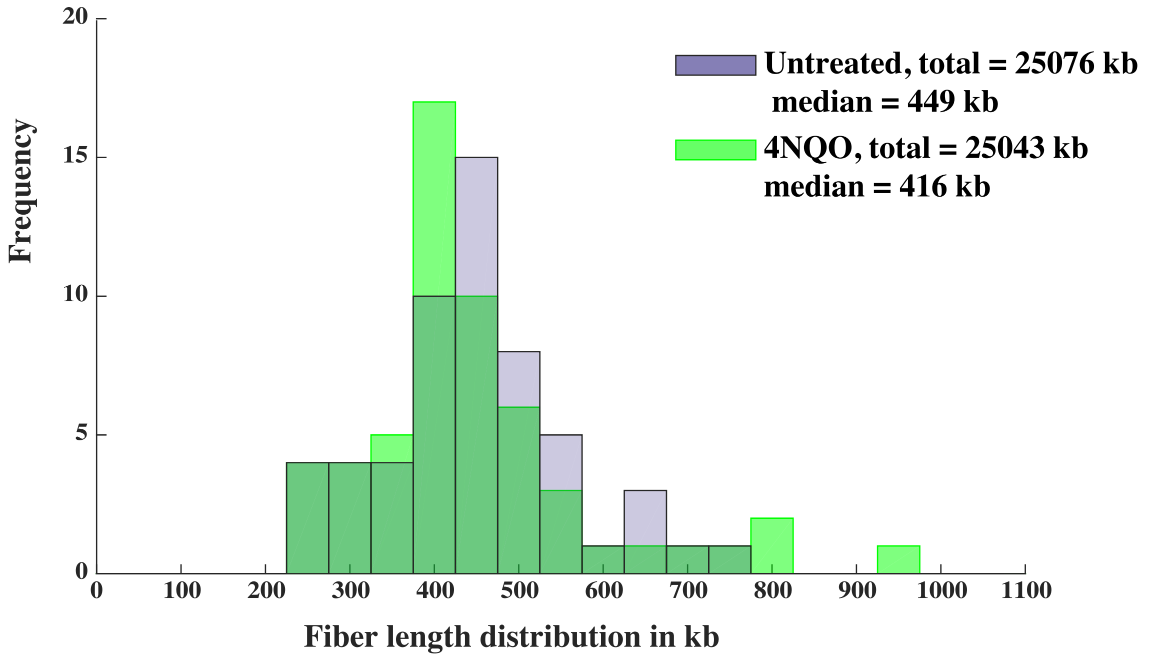

B **Wild-type - fiber length distribution in untreated and MMS sample**

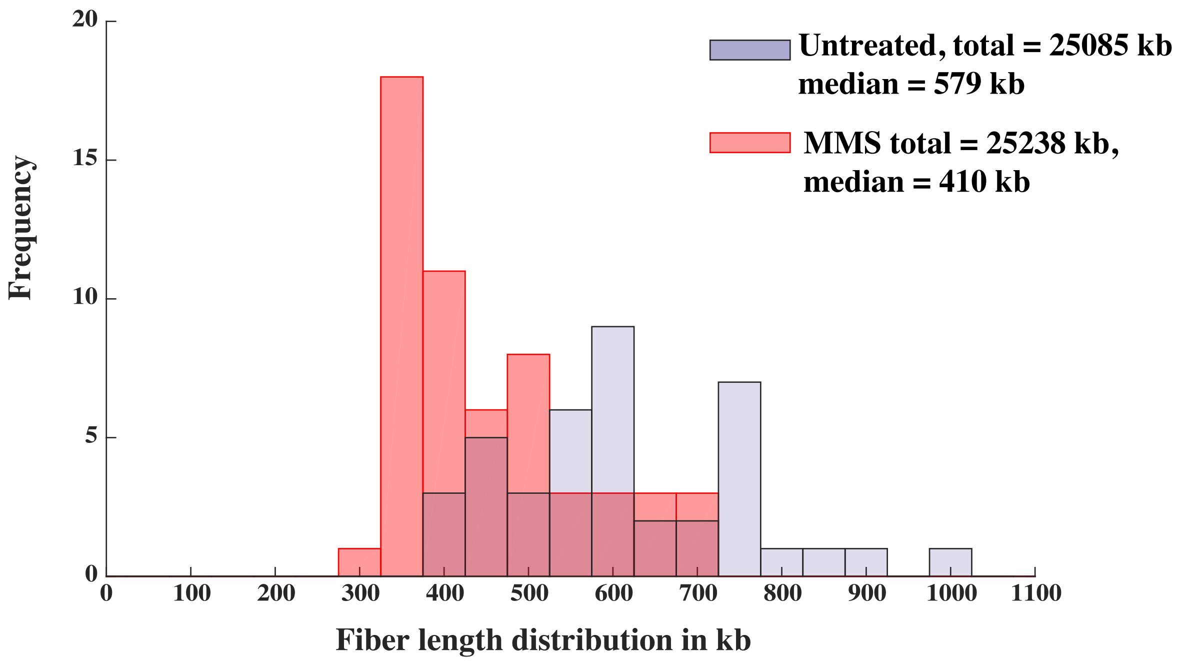

Figure S11: Fiber length distribution.
Length of fibers from wild-type 4NQO (A) and MMS (B) samples from a single experiment. Approximately 25Mb of total DNA was collected for each sample (Table S1).
