# Supplementary material for: Replication fork slowing and stalling are distinct, checkpoint-independent consequences of replicating damaged DNA
Source: PLoS Genet. 2017 Aug 14;13(8):e1006958. doi: 10.1371/journal.pgen.1006958 (PMC5570505; doi:10.1371/journal.pgen.1006958)
Supplement: S12 Fig — Cells were pulse labeled with 2 μM CldU for 5 minutes and chased with 20 μM IdU for varying lengths of time: 3, 4, 5, 6, 7, 8, 10, 14, 16 minutes. At least 100 forks were collected for each time point except for 3 and 4 minutes of IdU labeling for which only 11 and 44 forks were measured, respectively, due to lack IdU labeled forks at such short lengths of labeling period. (PDF) [file pgen.1006958.s012.pdf]

Figure S12

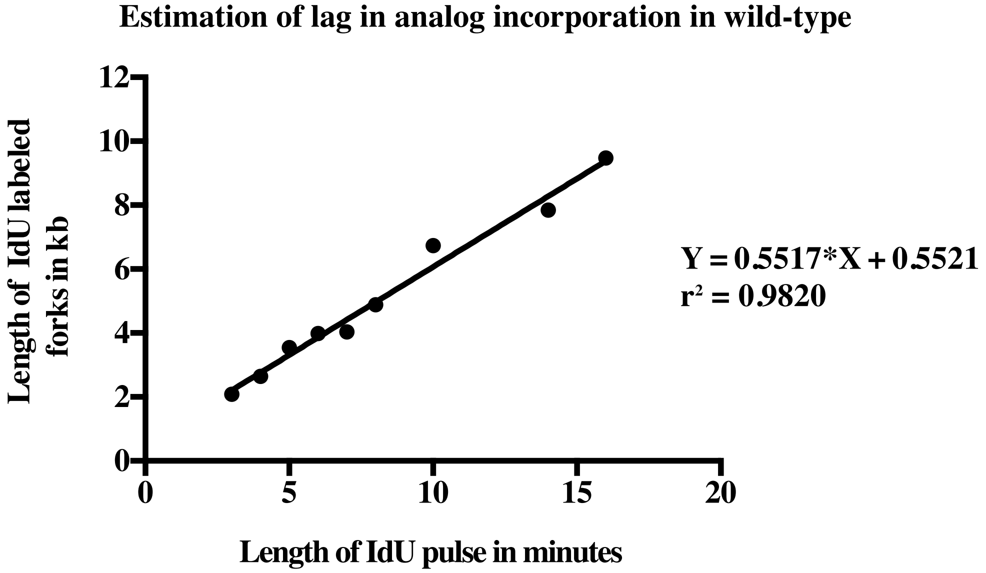

Figure S12: Estimation of lag in analog incorporation. Cells were pulse labeled with 2 μM CldU for 5 minutes and chased with 20 μM IdU for varying lengths of time: 3, 4, 5, 6, 7, 8, 10, 14, 16 minutes. At least 100 forks were collected for each time point except for 3 and 4 minutes of IdU labeling for which only 11 and 44 forks were measured, respectively, due to lack IdU labeled forks at such short lengths of labeling period.
