# Supplementary material for: Replication fork slowing and stalling are distinct, checkpoint-independent consequences of replicating damaged DNA
Source: PLoS Genet. 2017 Aug 14;13(8):e1006958. doi: 10.1371/journal.pgen.1006958 (PMC5570505; doi:10.1371/journal.pgen.1006958)
Supplement: S13 Fig — We re-analyzed the fibers considering stretching artifacts, which may lead to incorrect interpretation of analog incorporation patterns [86]. Ends of the fiber are most susceptible to such stretching artifacts and hence we ignored the labeled events occurring at the ends of the fiber and re-calculated the stall rate. However we do not see a significant change between the stall rate calculated from the whole fiber or after ignoring the labeled events occurring at the ends of the fiber. 4NQO and bleomycin experiments were done twice in each wild-type and cds1Δ. MMS experiments were done 5 and 3 times in wild-type and cds1Δ respectively. (PDF) [file pgen.1006958.s013.pdf]

Figure S13

Re-estimation of stall rate accounting for potential artefacts yields minimal variation

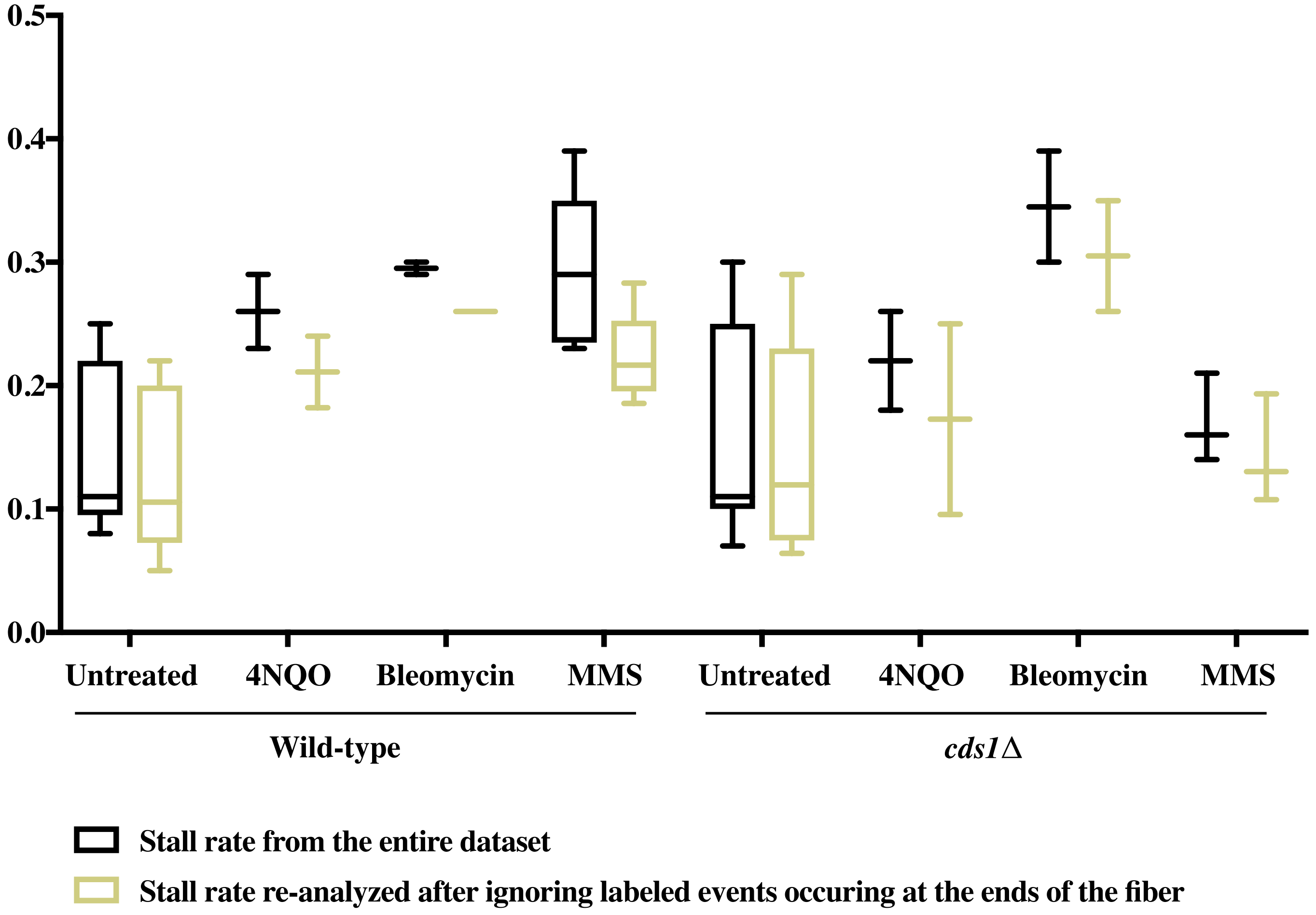

Figure S13: Re-estimation of stall rate accounting for potential artifacts yields minimal variation. We re-analyzed the fibers considering stretching artifacts, which may lead to incorrect interpretation of analog incorporation patterns [86]. Ends of the fiber are most susceptible to such stretching artifacts and hence we ignored the labeled events occurring at the ends of the fiber and re-calculated the stall rate. However we do not see a significant change between the stall rate calculated from the whole fiber or after ignoring the labeled events occurring at the ends of the fiber. 4NQO and bleomycin experiments were done twice in each wild-type and *cds1*Δ. MMS experiments were done 5 and 3 times in wild-type and *cds1*Δ respectively.
