# Supplementary material for: Replication fork slowing and stalling are distinct, checkpoint-independent consequences of replicating damaged DNA
Source: PLoS Genet. 2017 Aug 14;13(8):e1006958. doi: 10.1371/journal.pgen.1006958 (PMC5570505; doi:10.1371/journal.pgen.1006958)
Supplement: S14 Fig — Fork rate value was estimated from the second analog track continuing from the first analog as explained in the Methods section. (PDF) [file pgen.1006958.s014.pdf]

Figure S14

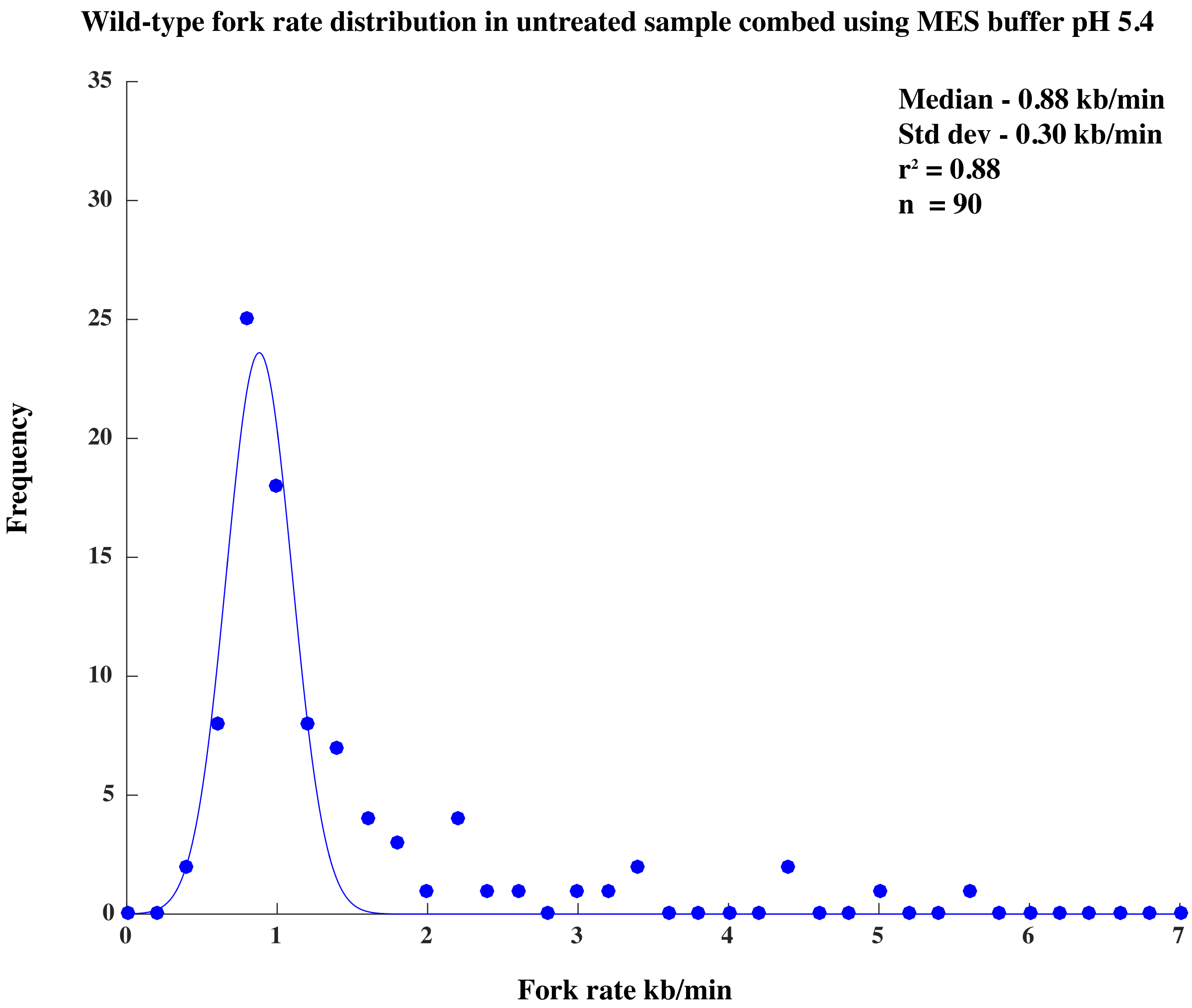

Figure S14: Fork rate distribution in wild-type untreated sample combed using MES buffer pH 5.4. Fork rate value was estimated from the second analog track continuing from the first analog as explained in the Methods section.
