# Supplementary material for: Replication fork slowing and stalling are distinct, checkpoint-independent consequences of replicating damaged DNA
Source: PLoS Genet. 2017 Aug 14;13(8):e1006958. doi: 10.1371/journal.pgen.1006958 (PMC5570505; doi:10.1371/journal.pgen.1006958)
Supplement: S15 Fig — A) Wild-type cells were synchronized and released into S phase with analog added at different concentrations—0.1 μM, 0.5 μM, 1 μM, 2 μM CldU—and chased with 20 μM IdU at 35 minutes after release or without any analog. B) The S-phase progression of cultures with and without analog addition used for combing experiments. 2 μM CldU analog was added at 50 minutes after release for 5 minutes and chased with 20 μM IdU for 10 minutes and the cells were harvested for combing at 65 minutes after release so as to have minimal effects of analog addition on replication kinetics. (PDF) [file pgen.1006958.s015.pdf]

Figure S15

A Addition of analog at the beginning of Sphase leads to slowing of S phase

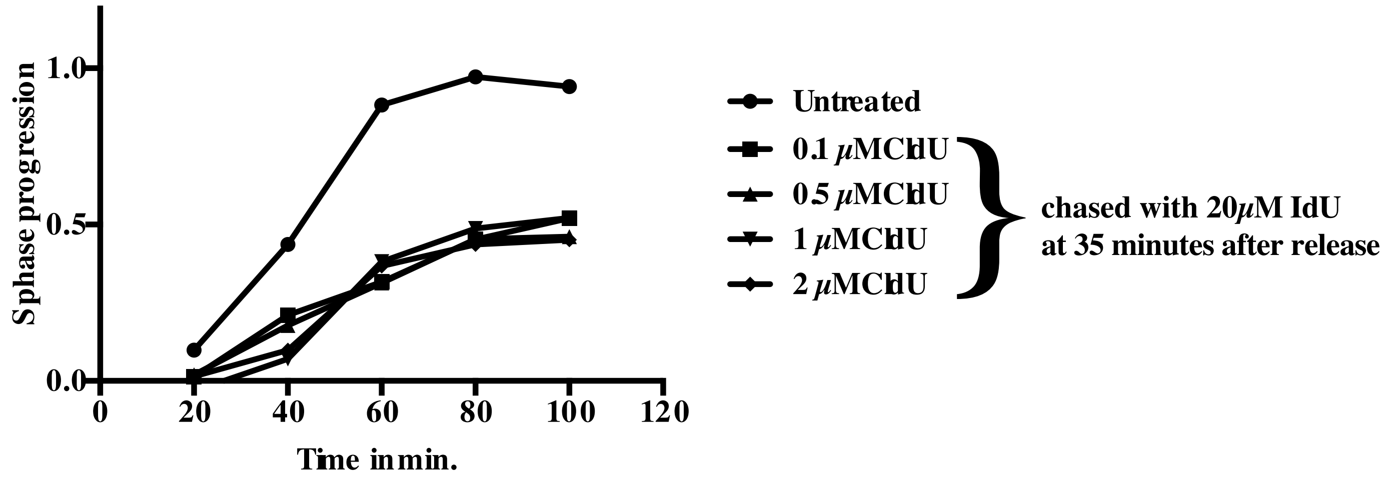

B Sphase progression in wild-type experiments used for combing with and without analog

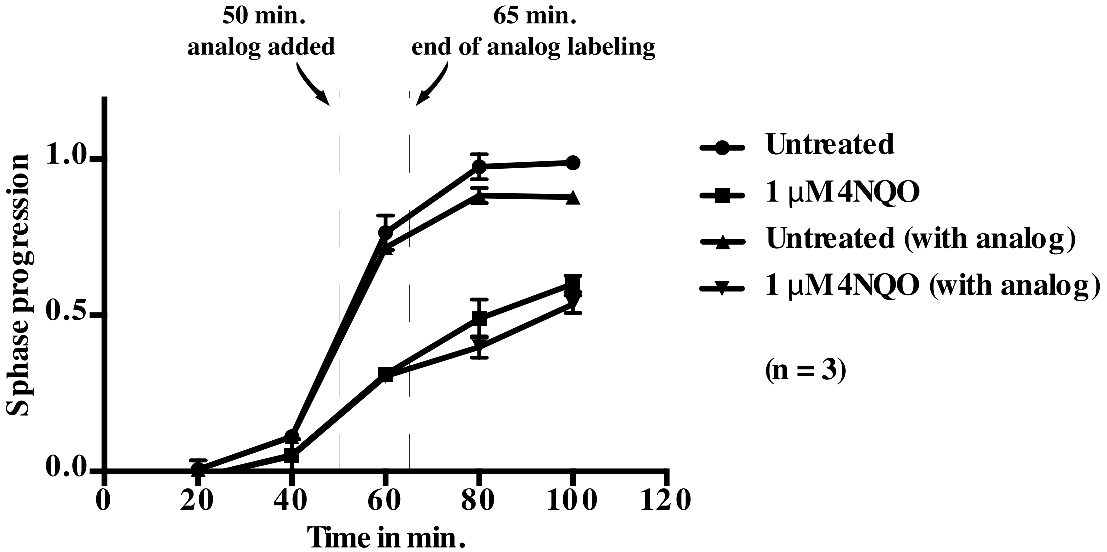

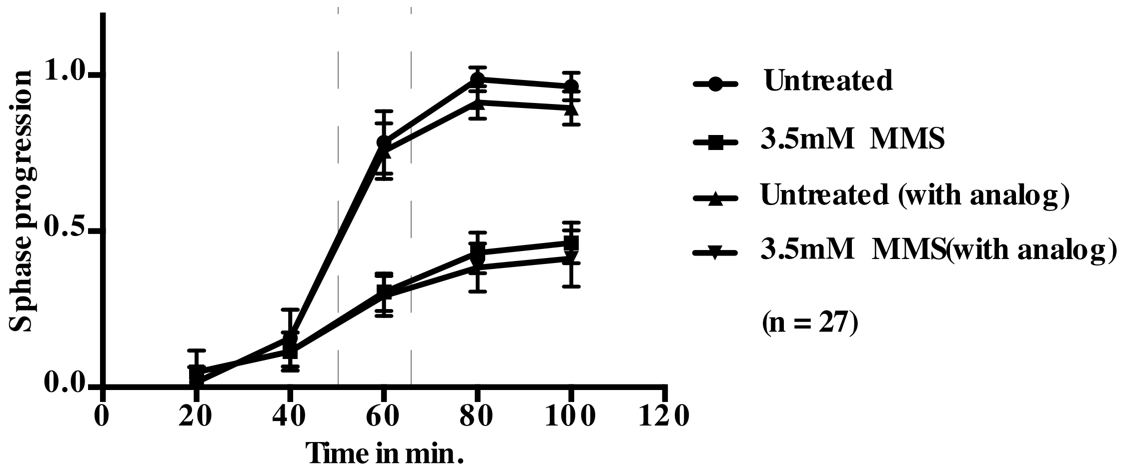

Figure S15: Effect of analog addition on replication kinetics. A) Wild-type cells were synchronized and released into S phase with analog added at different concentrations—0.1 μM, 0.5 μM, 1 μM, 2 μM CldU—and chased with 20 μM IdU at 35 minutes after release or without any analog. B) The S-phase progression of cultures with and without analog addition used for combing experiments. 2μ M CldU analog was added at 50 minutes after release for 5 minutes and chased with 20 μM IdU for 10 minutes and the cells were harvested for combing at 65 minutes after release so as to have minimal effects of analog addition on replication kinetics.
